# Differential Immunogenicity and Protective Efficacy Elicited by MTO- and DMT-Adjuvanted CMFO Subunit Vaccines against *Mycobacterium tuberculosis* Infection

**DOI:** 10.1155/2020/2083793

**Published:** 2020-09-04

**Authors:** Nadeem Ullah, Ling Hao, Yaqi Wu, Yandi Zhang, Qing Lei, Jo-Lewis Banga Ndzouboukou, Xiaosong Lin, Xionglin Fan

**Affiliations:** Department of Pathogen Biology, School of Basic Medicine, Tongji Medical College, Huazhong University of Science and Technology, 430030 Wuhan, China

## Abstract

Tuberculosis (TB) remains a major and global problem of public health. An effective TB subunit vaccine is urgently needed. Proper selection of the delivery system for the vaccine is crucial for inducing an appropriate immune response tailored to control the target pathogen. In this study, we compared the immunogenicity and protective efficacy of CMFO subunit vaccines against primary progressive TB in two different adjuvant systems: the MTO oil-in-water (O/W) emulsion composed of monophosphoryl lipid A (MPL), trehalose-6,60-dibehenate (TDB), and oil in water emulsion MF59 and the DMT liposome containing dimethyldioctadecylammonium bromide (DDA), monophosphoryl lipid A (MPL), and trehalose-6,60-dibehenate (TDB). Our results demonstrated that the DMT-adjuvanted CMFO could confer more significant protection against *M. tuberculosis* infection than the CMFO/MTO did in mice. In particular, the adjuvant DMT showed a stronger ability than the O/W emulsion to adjuvant CMFO subunit vaccine and enhanced protection, attributed to elicit Th1-biased responses, strong Th1/Th17 cytokine responses, and IFN-*γ*^+^ or IL-2^+^ T cell responses. Therefore, our findings demonstrate that the liposome delivery system shows more effectiveness to adjuvant TB subunit vaccine than O/W emulsion and highlight the importance of adjuvant formulation for the better efficacy of a protein vaccine.

## 1. Introduction

Bacillus Calmette Guerin (BCG), the only available vaccine for tuberculosis (TB), offers effective protection against severe forms of TB for children, whereas it fails to provide sufficient protection against adult pulmonary TB [1]. It is estimated by the World Health Organization (WHO) that the causative pathogen *Mycobacterium tuberculosis* causes about 10 million new TB cases, 2 billion latent TB infections (LTBIs), and 2 million deaths worldwide each year [2]. “End TB” strategy launched by the WHO is aimed at decreasing TB mortality by 95% until 2035 [3]. To achieve this great goal, it is urgent to develop a more effective TB vaccine.

Protein subunit vaccines have been developed increasingly based on definite components with good records of safety [4]. Due to the weak immunogenicity of the antigen itself, suitable adjuvants are required to enhance and/or shape the strength and type of antigen-specific immune responses induced by subunit vaccines [5]. Till now, there are only a few adjuvants in clinical use, such as aluminium salts, emulsions, liposomes, and polymeric particles [6]. These adjuvants with different structures and chemical compositions enable to induce different immune responses. Among them, oil-in-water (O/W) emulsions, such as MF59, AS03, and AF03, are known as safe and efficacious adjuvants in various vaccine products, already approved for human use or in clinical trials [7]. For example, MF59 has been tested in a variety of clinical trials for a wide array of antigens from influenza, HSV, HIV, HBV, HCV, and CMV [8]. Liposomes are alternative delivery systems to deliver a wide range of antigens as well as immunostimulants [9]. Liposomal adjuvant AS01 consisted of the agonist of toll-like receptor 4 (TLR4) monophosphoryl lipid A (MPLA) and QS21. AS01 adjuvanted the glycoprotein E subunit vaccine has been licensed for the prevention against adult VSV infection [10].

An addition of immunostimulants to adjuvant formulations might be a very promising strategy to promote appropriate protective immune responses following vaccination. Both O/W emulsions and liposomes alone have been evaluated in the context of additional immunostimulants in preclinical and preclinical trials. For instance, the inclusion of the TLR4 agonist E6020 in an MF59-adjuvanted meningococcus B vaccine enhanced serum and bactericidal titers in mice [11]. TB fusion protein ID93 in adjuvant of the O/W emulsion GLA-SE containing MPLA could elicit strong antigen-specific antibodies and cell-mediated immune responses, thus providing effective protection against TB [12]. Meanwhile, licensed liposome adjuvant AS01 has been confirmed to induce a Th1 response and IgG antibody. The other liposome dimethyldioctadecylammonium- (DDA-) based adjuvant CAF01 composed of mincle agonist trehalose-6,6′-dibehenate (TDB) could provoke a strong Th1/Th17 immune response in clinical trials and enhance protection against TB [13]. Previously, we constructed two novel adjuvants, the MTO O/W emulsion and the DMT liposome [14, 15], which were made up of the same immunostimulatory components such as MPLA and TDB, but with different delivery systems MF59 [14] or DDA liposome [15]. MTO- or DMT-adjuvanted different subunit vaccines could confer various protection against primary progressive TB in mouse models [14, 15]. However, it remains unclear whether different delivery systems may affect the efficacy of TB subunit vaccines, in order to develop next-generation TB vaccines. To this end, the immunogenicity and protective efficacy of MTO and DMT adjuvanted the same antigen CMFO subunit vaccines were compared in C57BL/6 mouse models.

## 2. Material and Methods

### 2.1. Ethical Statement

Animal experiments were performed in accordance with the guidelines of the Chinese Council on Animal Care. Research protocols were approved by Tongji School Committees on Biosafety and Animal Experimental Ethics.

### 2.2. Preparation of Adjuvanted Protein Subunit Vaccines

The multistage CMFO protein was purified and determined as previously described [16]. Each dose of the adjuvant DMT was composed of DDA 250 *μ*g, TDB 50 *μ*g, and MPLA 25 *μ*g (Avanti Polar Lipids, USA) and prepared by a thin lipid film method [15]. Each dose of the adjuvant MTO was prepared as previously described [14] and revised to contain the same dose of MPLA and TDB as the DMT and components of MF59 such as squalene oil (1%, *v*/*v*), Tween 80 (0.4%, *v*/*v*), and Span 85 (1%, *v*/*v*) (Sigma-Aldrich, MO, USA). 100 *μ*L of each CMFO solution (0.2 mg/mL) was thoroughly mixed with 100 *μ*L of the adjuvant DMT liposome or the MTO O/W emulsion to form different subunit vaccines, respectively.

### 2.3. Mice and Immunization

Specific pathogen-free, 6-8-week-old female C57BL/6 mice were purchased from Charles River (Beijing, China). Mice were immunized subcutaneously (s.c) twice at 3-week intervals with a 200 *μ*L dose of CMFO/DMT or CMFO/MTO. PBS, MTO, and DMT alone were used as negative controls.

### 2.4. Antigen-Specific IgG and Subclass Antibodies Detected by ELISA

10 weeks after immunization, serum samples were collected from each mouse in different groups. CMFO-specific IgG, IgG1, and IgG2a (Cat#151276, 133045, and 157720; Abcam, Cambridge, MA, USA) endpoint titers were detected by enzyme-linked immunosorbent assay (ELISA) as previously described [16]. The results were expressed as the mean (±SEM) log_10_ endpoint titers per group (*n* = 6).

### 2.5. Splenocytes Secreting CMFO-Specific Cytokines Measured by CBA

10 weeks after immunization, splenocytes were prepared and counted from each immunized mice in different groups. 5 × 10^6^ cells were seeded in each well of a 24-well microtiter plate in triplicate and restimulated with 10 *μ*g/mL CMFO for 72 h as previously described [15, 16]. 72 hours later, supernatant for each well was collected and a cytometric bead array (CBA) kit (BD Biosciences, USA) was used to detect the concentration of CMFO-specific Th1/Th2/Th17 cytokines containing IFN-*γ*, IL-2, TNF-*α*, IL-4, IL-6, IL-10, and IL-17 [16]. The results were expressed as the mean ± SEM (pg/mL) for each group (*n* = 6).

### 2.6. CMFO-Specific T Cell Responses Analyzed by Intracellular Cytokine Staining

Splenocytes or lung cells were prepared and counted from each mice in different groups as previously described, respectively [15, 16]. Cells were plated in triplicate at 5 × 10^6^ cells per well in a 24-well plate and incubated with CMFO (10 *μ*g/mL) and anti-CD28/CD49d (1 *μ*g/mL, eBioscience) overnight. RPMI 1640 medium used is a negative control. Cell responses were monitors through a cell stimulation cocktail (1 *μ*g/mL, eBioscience). Cells were stained for anti-CD4-APC-Cy7 (Cat#552051, BD Biosciences), anti-CD8*α*-BV510 (Cat#563068, BD Biosciences), anti-CD44-FITC (Cat#561859, BD Biosciences), anti-CD62L-PerCP-Cy5.5 (Cat#560513, BD Biosciences), and intracellular markers anti-IFN-*γ*-PE (Cat#554412, BD Biosciences) and anti-IL-2-APC (Cat#554429, BD Biosciences). Absolute number of IFN-*γ*^+^ and IL-2^+^ T cells, central memory T cells (T_CM_), and effector memory T cells (T_EM_) was determined by an LSRII multicolor flow cytometer (BD Biosciences) and analyzed through FlowJo software. The results were shown as the mean ± SEM per group (*n* = 6).

### 2.7. Challenged with Virulent *M. tuberculosis* H37Rv

10 and 20 weeks after immunization, mice were challenged intranasally (i.n.) with approximately 100 CFU of *M. tuberculosis* H37Rv strain, respectively. Four weeks postchallenge, six mice in each group were sacrificed and the lung and spleen were aseptically removed, respectively. Bacterial load (log_10_ CFU) per organ was enumerated. After HE and acid-fast staining, lung histopathological changes were assessed as previously described [15, 16].

### 2.8. Statistical Analysis

Statistical analysis was performed by using GraphPad Prism 6 and SPSS version 22.0 software. The Student *t*-test and ANOVA test (one-way or two-way) were used to measure the difference between two groups or various groups, respectively. The statistic difference was considered significant when a value of *p* < 0.05.

## 3. Results

### 3.1. Differential Antibody Responses to CMFO Were Provided by MTO- and DMT-Adjuvanted CMFO Subunit Vaccines

**Figure 1:**
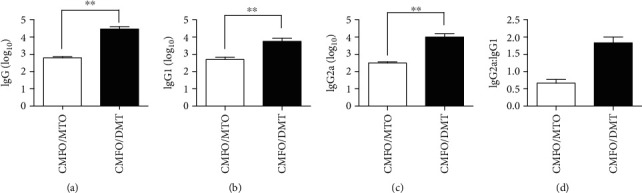
CMFO-specific antibody responses in different immunized mice (*n* = 6). Mice were vaccinated with PBS, MTO, DMT, CMFO/MTO, and CMFO/DMT. 10 weeks later, CMFO-specific antibodies IgG (a), IgG1 (b), and IgG2a (c) in the serum of each mouse were tittered by ELISA. The results were shown as the mean (± SEM) log_10_ endpoint titers. (d) The ratio of IgG2a : IgG1 in the different groups of vaccinated mice. ^∗∗^*p* < 0.001.

To compare the immunogenicity between CMFO/MTO and CMFO/DMT, CMFO-specific antibodies in the serum of each mouse in different groups were determined by ELISA (Figure 1). As expected, there was not any CMFO-specific IgG, IgG1, and IgG2a detected in PBS, or adjuvants DMT and MTO alone control mice (data not shown). Interestingly, CMFO/DMT-vaccinated mice elicited much higher antibody titers of IgG, IgG1, and IgG2a against CMFO than CMFO/MTO did, respectively. In addition, the ratio of IgG2a/IgG1 response to the antigen CMFO indicated that CMFO/DMT-vaccinated mice induced a Th1-biased response and a mixed Th1 and Th2 response in the CMFO/MTO group.

### 3.2. Differential Cytokine Responses to CMFO Were Induced by MTO- and DMT-Adjuvanted CMFO Subunit Vaccines

**Figure 2:**
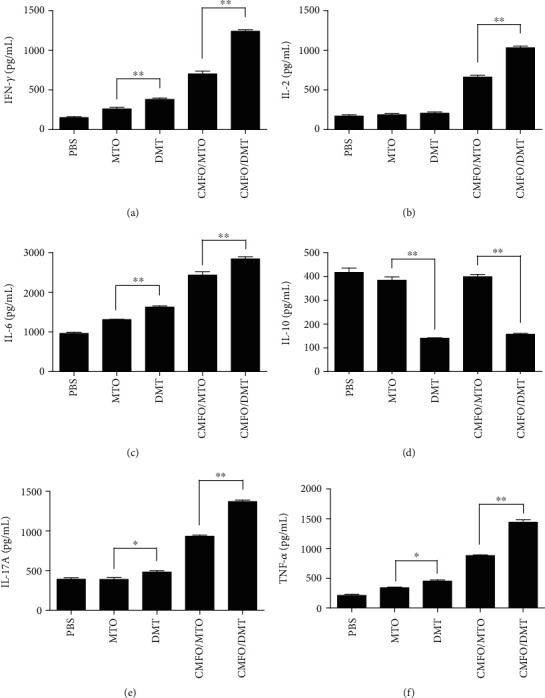
The levels of CMFO-specific cytokines secreted by splenocytes (*n* = 6). 10 weeks after immunization, 5 × 10^6^ splenocytes from different immunized mice were restimulated with 10 *μ*g/mL CMFO for 72 h. The supernatants were collected from each well, and the levels of CMFO-specific Th1/Th2/Th17 cytokines containing IFN-*γ* (a), TNF-*α* (b), IL-2 (c), IL-10 (d), IL-6 (e), and IL-17A (f) were detected by a CBA kit. The results were expressed as the mean ± SEM (pg/mL). ^∗^*p* < 0.05 and ^∗∗^*p* < 0.001. The concentration of IL-4 in each group was less than 1 pg/mL, and data were not shown.

10 weeks after immunization, CMFO-specific cytokines in the supernatant of splenocytes from different vaccinated mice were detected by a CBA kit (Figure 2). Among all groups, splenocytes from PBS control mice produced the lowest levels of detected cytokines except IL-10. Interestingly, MTO and DMT alone induced higher levels of IFN-*γ*, TNF-*α*, and IL-6 than the PBS control. Moreover, higher levels of CMFO-specific IFN-*γ*, TNF-*α*, IL-6, and IL-17A were elicited in DMT control mice than that of the MTO group. Correspondingly, CMFO in adjuvants of MTO- or DMT-vaccinated mice produced significantly higher levels of IFN-*γ*, TNF-*α*, IL-2, IL-6, and IL-17A than PBS or adjuvant controls, respectively. More importantly, CMFO/DMT-vaccinated mice produced higher levels of IFN-*γ*, TNF-*α*, IL-2, IL-6, and IL-17A. In addition, there was no statistical difference in the concentration of IL-10 between PBS, MTO alone, or MTO-adjuvanted CMFO-vaccinated mice. The concentration of the IL-4 was lower than 1 pg/mL in all groups (data not shown). Together, these data confirm that the adjuvant DMT has a stronger ability to induce more significant Th1 and Th17 cytokines than the MTO O/W emulsion.

### 3.3. Differential T Cell Responses to CMFO Were Elicited by MTO- and DMT-Adjuvanted CMFO Subunit Vaccines

**Figure 3:**
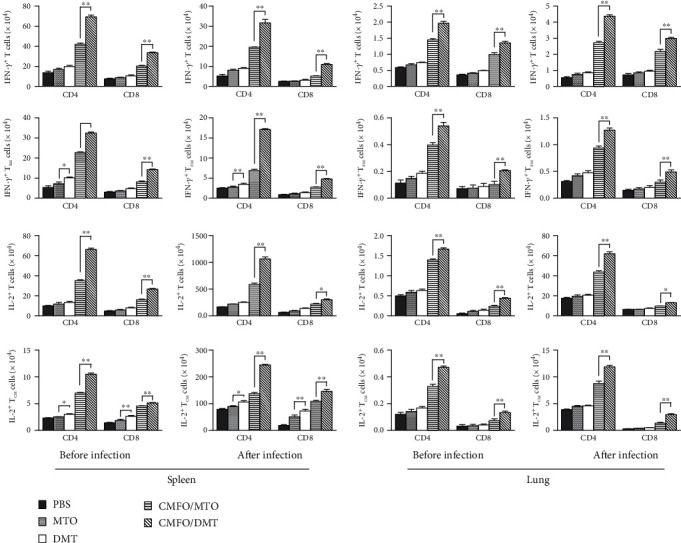
CMFO-specific T cells in the spleen and lung of different vaccinated mice before and after exposure (*n* = 6). 10 weeks after vaccination and 4 weeks postchallenge, splenocytes and lung cells were prepared from each mice and counted, respectively. The absolute numbers of CMFO-specific IFN-*γ*^+^ or IL-2^+^ CD4^+^ and CD8^+^ T cells, or T_CM_ cells secreting IL-2 and T_EM_ cells secreting IFN-*γ* per spleen and per lung were identified by intracellular cytokine staining and a multicolor flow cytometer. The results were shown as the mean ± SEM. ^∗^*p* < 0.05 and ^∗∗^*p* < 0.001.

To analyze the effects of adjuvant and delivery system on the protection, the number of CMFO-specific T cells in the spleen and the lung from different vaccinated mice was also detected by FACS (Figure 3). As expected, PBS control mice had the lowest number of IFN-*γ*^+^ or IL-2^+^ T cells, IFN-*γ*^+^ T_EM_ or IL-2^+^ T_CM_ cells of all groups. Importantly, much more of IFN-*γ*^+^ or IL-2^+^ T cells, IFN-*γ*^+^ T_EM_ cells and IL-2^+^ T_CM_ cells were induced in the spleen and the lung DMT-adjuvanted CMFO subunit-vaccinated mice than the CMFO/MTO group, whatever before and after infection. When compared with the levels of before infection, more IL-2^+^ CD4^+^ T cells and IL-2^+^ CD4^+^ T_CM_ cells were elicited in the spleen and the lung of the CMFO/DMT group after infection with *M. tuberculosis*. In addition, DMT alone induced much more levels of CMFO-specific IFN-*γ*^+^ CD4^+^ T_EM_ cells and IL-2^+^ CD4^+^ T_CM_ cells in the spleen than MTO control mice before infection.

### 3.4. Differential Protective Efficacies Were Conferred by MTO- and DMT-Adjuvanted CMFO Subunit Vaccines

**Figure 4:**
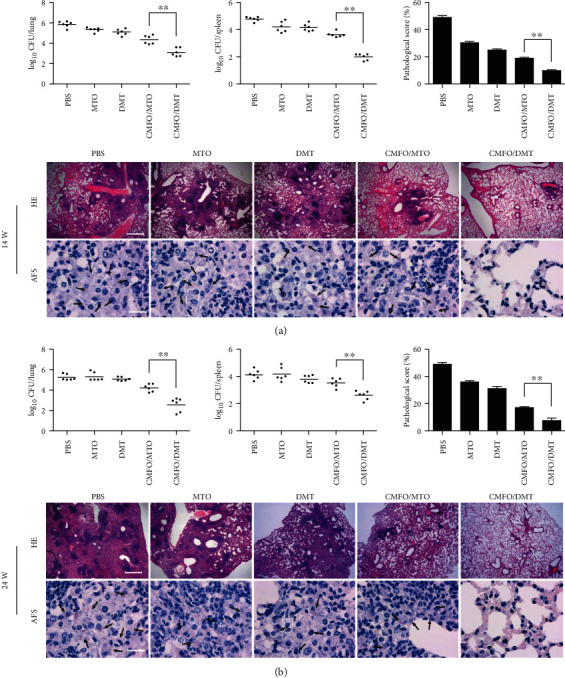
Short-term and long-term protection efficacies between different vaccinated groups. Vaccination and challenge schedule. At the 14th (a) and 24th weeks (b), bacterial load in the lung and spleen per organ was enumerated. The results were shown as the mean ± SEM log_10_ CFU (*n* = 6). Representative histopathological photographs of the lung and lung histopathological score (*n* = 3) from short-term and long-term experiments were also provided. HE staining, scale bar = 500 *μ*m and AF staining, scale bar = 20 *μ*m. Arrowheads indicate AF positive bacteria. ^∗∗^*p* < 0.001.

10 weeks and 20 weeks after immunization, mice in different groups were challenged with virulent *M. tuberculosis* H37Rv to assess short-term (Figure 4(a)) and long-term (Figure 4(b)) protective efficacies, respectively. Of all groups, PBS control mice had the highest bacterial load in the lung and spleen during the experimental periods. Interestingly, stronger short-term and long-term protection was obtained in CMFO/MTO- or CMFO/DMT-vaccinated mice than the PBS control. More importantly, DMT in adjuvant of CMFO-vaccinated mice demonstrated the most significant inhibition of bacterial growth in both organs of all groups. In addition, bacterial growth in both organs of MTO- or DMT-alone-vaccinated mice was mildly inhibited only at 14 weeks, when compared to the PBS control.

The results of lung tissue section stained with hematoxylin-eosin (HE) or acid-fast (AF) staining further supported differential bacterial loads in the lung of different groups. Control mice such as PBS, MTO, and DMT alone demonstrated the most serious histopathology with extensive fibrosis and pulmonary alveolitis of all groups with the highest histopathological scores, and AF-positive bacilli were found throughout the whole lung section of these control mice. In contrast, pulmonary lesions and inflammation of mice in the CMFO/MTO groups were substantially reduced and a few AF-positive bacilli were detected in the alveolar tissue. Remarkably, CMFO/DMT-vaccinated mice exhibited had the fewest lesions and the lowest scores of all groups, with no AF-positive bacilli found in the section.

## 4. Discussion

Although adjuvants play critical roles in the development of an effective TB subunit vaccine [6, 16], the effect of adjuvant delivery systems on the efficacy of TB subunit vaccines remains to be investigated [6]. To this end, we compared the immunogenicity and protective efficacy of CMFO subunit vaccines in two adjuvants of DMT and MTO with different delivery systems against primary progressive TB in C57BL/6 mice in this study. Our results demonstrated that CMFO/DMT could confer more significant protection against *M. tuberculosis* infection than CMFO/MTO in mice, as demonstrated by a reduced bacterial load in the lungs and spleens, and less lung pathological changes. In particular, the adjuvant DMT showed a stronger ability than O/W emulsion to adjuvant CMFO subunit vaccine and enhanced protection, attributed to elicit Th1-biased responses, strong Th1/Th17 cytokine responses, and IFN-*γ*^+^ or IL-2^+^ T cell responses. Therefore, our findings confirm that the liposome delivery system is more effective to adjuvant TB subunit vaccine than O/W emulsion.

TDB alone activates mincle and FcRgammaSyk-Card9 pathways to trigger MyD88-dependent Th1 and Th17 responses [17]. MPLA can be recognized by TLR4 on the surface of APCs and stimulates NF-*κ*B cells through MyD88- and TRIF-dependent pathways, thus inducing a Th1-biased immune response [18]. MF59 adjuvant has good stability and can promote antigen-presenting cells (APCs) to process and present antigens [19]. Previous studies demonstrated that MF59 could induce a mixed Th1 and Th2 response in influenza, Middle East respiratory syndrome, or TB-infected mouse models [11, 14, 20, 21]. MTO-adjuvanted A1D4 subunit vaccine showed insufficient ability to induce single and multifunctional IL-2^+^ T cells, which thus resulted in the inferior protective efficacy against *M. tuberculosis* infection to the BCG vaccine [14]. A Th2-biased adjuvant (SE) fails to protect mice or guinea pigs against *M. tuberculosis* infection [22]. In line with these findings, MTO-adjuvanted CMFO also induced a mixed Th1 and Th2 response and insufficient T cell responses. Liposomes also can be used as the delivery system of vaccines and improve their effectiveness for different diseases, such as dengue fever, TB, influenza, and malaria [23–26]. Consistent with previous studies [15–18], liposomal-adjuvanted subunit vaccine CMFO/DMT induced a Th1-biased response as well as Th1/Th17 cytokines in this study. Moreover, T_CM_ cells and T_EM_ cells are effective biomarkers correlated with vaccine-induced protection against TB [16]. The domination of IL-2^+^ CD4^+^ T_CM_ cells and IFN-*γ*^+^ CD4^+^ T_EM_ cells in the infected lung might be more meaningfully associated with CMFO/DMT subunit vaccine-induced protection.

Proper selection of the delivery system for the vaccine is crucial for inducing an appropriate immune response tailored to control the target pathogen. Liposome as the delivery system of the vaccine can induce a Th1-biased response, while O/W emulsion elicits a mixed Th1 and Th2 response. DMT liposome is more suitable to use as the adjuvant of TB vaccine candidates than MTO O/W emulsion, which warrants for further preclinical studies to accelerate the development of TB subunit vaccines.

## Data Availability

The original data used to support the findings of this study are available from the corresponding author upon request.

## Abbreviations

CMFO: Rv2875-Rv3044-Rv2073c-Rv0577
DMT: DDA-MPLA-TDB
MTO: MPLA-TDB-MF59
TB: Tuberculosis
BCG: Bacillus Calmette-Guerin
DDA: Dimethyldioctadecylammonium
MPLA: Monophosphoryl lipid A
TDB: Trehalose-6,6′-dibehenate
CBA: Cytometric bead array
FACS: Fluorescence-activated cell sorting
TLR4: Toll-like receptor 4
O/W: Oil in water.
